# Ketogenic-Mimicking Diet as a Therapeutic Modality for Bipolar Disorder: Biomechanistic Rationale and Protocol for a Pilot Clinical Trial

**DOI:** 10.3390/nu15133068

**Published:** 2023-07-07

**Authors:** Jeffrey L. B. Bohnen, Travis P. Wigstrom, Alexis M. Griggs, Stiven Roytman, Noah Paalanen, Hailemicael A. Andrews, Nicolaas I. Bohnen, Jacob J. H. Franklin, Melvin G. McInnis

**Affiliations:** 1 Department of Neurology, University of Michigan, Ann Arbor, MI 48109, USA; 2 University of Michigan Medical School, Ann Arbor, MI 48109, USA; 3 Neurology Service and GRECC, VA Ann Arbor Healthcare System, Ann Arbor, MI 48105, USA; 4 Morris K. Udall Center of Excellence for Parkinson’s Disease Research, University of Michigan, Ann Arbor, MI 48109, USA; 5 Parkinson’s Foundation Research Center of Excellence, University of Michigan, Ann Arbor, MI 48109, USA; 6 Department of Radiology, University of Michigan, Ann Arbor, MI 48109, USA; 7 Department of Psychiatry, University of Michigan, Ann Arbor, MI 48109, USA; 8 Heinz C. Prechter Bipolar Research Program, University of Michigan, Ann Arbor, MI 48109, USA

**Keywords:** ketosis, ketone ester, metabolic psychiatry, mitochondria, mood, metabolism, network stability

## Abstract

There is growing interest in the investigation of ketogenic diets as a potential therapy for bipolar disorder. The overlapping pharmacotherapies utilized for both bipolar disorder and seizures suggest that a mechanistic overlap may exist between these conditions, with fasting and the ketogenic diet representing the most time-proven therapies for seizure control. Recently, preliminary evidence has begun to emerge supporting a potential role for ketogenic diets in treating bipolar disorder. Notably, some patients may struggle to initiate a strict diet in the midst of a mood episode or significant life stressors. The key question addressed by this pilot clinical trial protocol is if benefits can be achieved with a less restrictive diet, as this would allow such an intervention to be accessible for more patients. Recent development of so-called ketone esters, that once ingested is converted to natural ketone bodies, combined with low glycemic index dietary changes has the potential to mimic two foundational components of therapeutic ketosis: high levels of ketones and minimal spiking of glucose/insulin. This pilot clinical trial protocol thus aims to investigate the effect of a ‘ketogenic-mimicking diet’ (combining supplementation of ketone esters with a low glycemic index dietary intervention) on neural network stability, mood, and biomarker outcomes in the setting of bipolar disorder. Positive findings obtained via this pilot clinical trial protocol may support future target engagement studies of ketogenic-mimicking diets or related ketogenic interventions. A lack of positive findings, in contrast, may justify a focus on more strict dietary interventions for future research.

## 1. Introduction

Bipolar disorder is a disabling chronic condition that poses unique challenges to treatment [1,2,3,4]. Treatment of bipolar depression is less well investigated than treatment of unipolar depression, with antidepressant treatment bearing additional risks of evoking mania, destabilizing mood, and inducing dysphoria or potential suicidality in the context of bipolar disorder [5,6,7,8]. On the other side of the spectrum, it is not uncommon for patients to exemplify inconsistent (or nonexistent) adherence to a mood stabilizer regimen for the treatment of mood elevation. Among patients with bipolar disorder, doubts about the need for medications as well as concerns for adverse effects have been cited as drivers of markedly high rates of medication nonadherence, which may approach 50% (or potentially as high as 69% by some estimates) [9,10].

With a patient-centered approach to care, global functioning represents another important consideration alongside symptomatic relief. In a study that followed patients for 2–4 years after their first hospitalization for mania, 28% of patients remained symptomatic and 57% did not return to their prior level of functioning [11]. While pharmacological breakthroughs have provided undeniable value and saved countless lives, these findings suggest the possibility that existing therapeutic paradigms may be further improved upon as our understanding of this disorder evolves.

What remains unknown is how exactly clinical practice can be further advanced, particularly in light of an evolving pathophysiological understanding of bipolar disorder. While emerging evidence supports a potential role for metabolic interventions such as a ketogenic diet in the treatment of this complex disorder, this possibility raises further questions related to policy challenges and insurance reimbursement hurdles affecting the delivery of care for nutrition-related interventions. Whereas nutrition-related interventions offer the possibility of transformative pleiotropic benefits, they are also considered to be more complex to incorporate into research methodology and clinical practice as compared to pharmaceutical treatments. Adherence represents a key consideration in psychiatric populations, providing an impetus to consider interventions that may be more easily implemented for patients who struggle with self-discipline or are in the midst of significant life stressors. Drawing on this line of reasoning, investigation of exogenous ketone supplements may reveal strategies for improving the accessibility of therapeutic ketosis. To the authors’ best knowledge, there is not a single clinical study investigating exogenous ketone supplementation in the setting of bipolar disorder to date.

Importantly, the effects of exogenous ketone supplementation are widely known to be modulated by dietary intake (in particular, glycemic/insulin spiking). Exogenous ketone supplementation without any other dietary changes may thus confer limited benefit depending on the clinical context. As a potential middle ground, combining exogenous ketone supplementation with low glycemic index dietary changes may mimic two foundational components of strict ketogenic diets: namely, high levels of ketones and minimal glycemic/insulin spiking. This pilot clinical trial protocol thus aims to investigate the biomechanistic effects of exogenous ketone supplementation combined with low glycemic index dietary changes in the setting of bipolar disorder.

## 2. Biomechanistic Rationale

Although the exact mechanisms underlying bipolar disorder remain unclear, it is evident that agents implicated in neuronal membrane stability are effective in mitigating symptoms [12,13,14,15,16]. The overlapping pharmacotherapies utilized for both bipolar disorder and seizures suggest that a mechanistic overlap may exist between these conditions. A corollary hypothesis derived from this possibility is that treatments with proven efficacy in the realm of seizure management may also prove to be beneficial for patients with bipolar disorder. Interestingly, one of the most time-proven approaches to seizure control is the induction of ketosis, with Hippocrates himself documenting fasting as a remedy for seizures [17]. In 1921, Dr. Russell Wilder at the Mayo Clinic postulated that the benefits of fasting may be dependent on high levels of ketones in the blood, speculating that similar benefits may be derived if ketonemia was achieved by other means [18]. Hence, the ketogenic diet was born, with breakthroughs that followed in the treatment of seizures.

With these considerations in mind, the ketogenic diet may also offer a promising novel therapeutic option for patients suffering from bipolar disorder. This hypothesis has been supported by limited preliminary data. In a study by Phelps et al. [19] two women with bipolar disorder were able to maintain the ketogenic diet for at least two years. Both women tolerated the diet well and achieved mood stabilization exceeding that attained with medication, reporting subjective improvement distinctly related to ketosis. Ultimately, both patients were able to discontinue mood-stabilizing medications and maintain mood stability with the ketogenic diet as a stand-alone intervention. In a data mining analysis comparing hundreds of anecdotal reports from online bipolar disorder forums, Campbell et al. [20] found that reports of symptomatic remission were significantly higher for the ketogenic diet in comparison to other health-conscious diets (with approximately 73% of reports endorsing some degree of mood stabilization with the ketogenic diet, as compared to approximately 54% of reports endorsing some degree of mood stabilization with other health-conscious diets). More recently, a retrospective analysis that included 13 patients with bipolar disorder reported significant clinical improvements when the ketogenic diet was utilized in an inpatient setting (depressive symptom severity was reduced by over 60%, with concordant improvements in clinical global impression) [21].

Conceptually, there are other reasons to investigate ketogenic interventions as a potential treatment modality for patients with bipolar disorder. Accumulating evidence has provided increasing support for the hypothesis that mitochondrial dysfunction may be an underlying feature of bipolar disorder [22,23,24]. Notably, mitochondrial dysfunction has also been implicated in the pathogenesis of epilepsy [25]. Considering the profound mitochondrial effects of ketogenic interventions, it is plausible that improved bioenergetic efficiency attained via therapeutic ketosis may promote stabilization of cellular functioning and, thereby, clinical improvement. Notably, the Na^+^/K^+^ ATPase, responsible for regulating neuronal membrane potential, represents a major beneficiary of bioenergetic availability. By some estimates, this ATPase consumes nearly half of the ATP in the brain [26]. In a setting of Na^+^/K^+^ ATPase hypofunctionality, sodium would be expected to accumulate within neurons, altering resting potential (and, thereby, altering patterns of neuronal excitability). Interestingly, individuals with bipolar disorder have been observed to exhibit decreased Na^+^/K^+^ ATPase activity and increased intracellular sodium concentrations [27]. Moreover, mood stabilizers shown to be effective in bipolar disorder converge upon a shared mechanism: reduction of intracellular sodium concentrations [14,28,29,30]. Similarly, ketogenic interventions may also converge upon this mechanism. In addition to powering the Na^+^/K^+^ ATPase by supporting mitochondrial energetic output, therapeutic ketosis may also lower intracellular sodium by inducing a mildly acidotic state, as extracellular protons will be exchanged for intracellular sodium [31,32,33]. An acidic neuronal environment also reduces neuronal excitability via a variety of other mechanisms, including modulation of calcium channel conductance [34]. Furthermore, a ketogenic diet may elevate GABA/glutamate ratios, an effect mediated by the gut-brain axis [35,36,37]. Modulation of neuronal excitability is thus a plausible mechanism attributable to ketogenic interventions, particularly in light of the clinical outcomes achieved by the ketogenic diet in the realm of epilepsy.

Altered patterns of synaptic plasticity have also been identified in the setting of bipolar disorder, bearing implications for interventions that modulate synaptic plasticity [38]. Animal models have demonstrated that ketone bodies are capable of stimulating brain-derived neurotrophic factor (BDNF) production [39,40], converging with evidence in human research [41,42,43]. BDNF is widely known as a vital modulator of synaptic plasticity.

Looking beyond the level of the synapse, a growing body of functional neuroimaging research has revealed that bipolar disorder is characterized by abnormalities in neural network functional connectivity. In particular, altered interactions between the amygdala and prefrontal regions have been implicated in bipolar disorder [44]. Via an elegant fMRI analysis, Mujica-Parody et al. [45] found that the ketogenic diet has a stabilizing effect on neural network functional connectivity. Moreover, exogenous ketone esters also demonstrated a stabilizing effect on neural network functional connectivity, even if consumed in the setting of a regular diet. Theoretically, the stabilization of neural networks may also bear implications for the management of bipolar disorder. This line of reasoning would be consistent with patterns of altered neuronal excitability identified in the setting of bipolar disorder [46], as altered patterns of spreading activation would be an expected downstream effect of altered neuronal excitability.

Furthermore, with a growing body of evidence demonstrating intersections between neuroinflammatory processes and psychiatric disorders, it is plausible that the anti-inflammatory properties of ketone bodies may prove to be therapeutic for patients suffering from bipolar disorder [47,48,49]. Neuroinflammatory processes have been implicated in the pathogenesis of the bipolar disorder, as demonstrated via markers of excitotoxicity and neuroinflammation mediated by the IL-R cascade [50]. Ketone bodies have been shown to inhibit activation of the NF-κB inflammasome (a key orchestrator of this signaling cascade) [51,52].

Intertwined with all of these factors, a final consideration is the role of epigenetics in the pathophysiology of bipolar disorder. In light of the clear impact of environmental factors on this episodic disorder, it is plausible that fluctuations in epigenetic expression may underlie shifts in symptomatology. Of particular relevance to this trial is the finding that ketone metabolism drives activation of sirtuins (which have been referred to as “the guardians of the genome” given their central role in epigenetic regulation), in addition to modulating epigenetic expression via a variety of other mechanisms [53,54,55,56].

## 3. Methods

### 3.1. Study Design: Overview

At the time of this writing, many scientific questions remain unanswered with a clear need for further research. In designing a pilot study, it is important to consider the distinction between a strict ketogenic diet and exogenous ketones, the latter of which can be consumed in a supplementary manner while maintaining a regular or modified diet. Clinical benefits derived from the ketogenic diet are likely attributable to a combination of low glycemic index and the direct (and indirect) metabolic/signaling effects of ketone bodies themselves. However, the proportional contribution from each of these components is not fully understood and likely varies based on the clinical setting. While a strict ketogenic diet encapsulates the entirety of these potential contributory components, it also sets a high bar for adherence in a population that already struggles with treatment adherence. In order to optimize ease of adherence, we propose supplementation of exogenous ketone esters combined with a low glycemic index diet for this pilot study, which will track clinical outcomes and global functioning metrics alongside metabolic markers. Moreover, given the stabilizing effects on neural networks demonstrated with exogenous ketone ester supplementation, the protocol features collection of baseline and follow-up fMRI data to assess neural network stability before and after the intervention. Combining clinical assessments, metabolic markers, and functional neuroimaging data into a multifaceted analysis will provide a comprehensive window into the dysregulation underlying bipolar disorder and the mechanisms by which this metabolic intervention may affect outcomes for our patients.

### 3.2. Experimental Intervention

The overarching goal of this open-label, exploratory pilot study is to explore biomechanistic effects of exogenous ketone supplementation combined with a low glycemic diet (in effect, a ‘ketogenic-mimicking diet’) for patients with bipolar disorder. This represents a less restrictive metabolic intervention compared to a strict ketogenic diet while replicating two foundational elements of therapeutic ketosis: low levels of glycemic/insulin signaling and high levels of circulating ketones. In addition to assessments of mood stability and global functioning, we will obtain functional neuroimaging data to better characterize the biomechanistic effects of this intervention.

### 3.3. Outcome Measures

Primary outcome measure:

- Neural network stability as ascertained via functional magnetic resonance imaging (fMRI)

Secondary outcome measures:

- Neural uptake of ketone bodies as ascertained via magnetic resonance spectroscopy (MRS)
- Clinical assessments and global functioning metrics (see Table 1)
- Laboratory assessments (see Table 2)
- Continuous glucose monitoring (CGM) glycemic trends
- ß-hydroxybutyrate (ß-HB) blood level (as ascertained via Keto-Mojo™ monitoring device)
- Sleep quality (as ascertained via Oura™ biometric ring)

Primary Aim: To compare fMRI neural network stability before and after a low glycemic index dietary intervention combined with open-label supplementation of exogenous ketones, namely bis hexanoyl (R)-1,3-butanediol (available over the counter as an athletic performance and health supplement called Juvenescence ‘Metabolic Switch^®^’ powder and also referred to as ‘C6 ketone di-ester’) in a biomechanistic pilot study in individuals with bipolar disorder (*n* = 34 [please see Supplementary Materials for sample size calculation]). While a comprehensive low glycemic index diet may also be considered to augment the effects of exogenous ketone supplementation, for simplicity and ease of adherence the low glycemic index intervention in this protocol will be comprised of four simple dietary changes as follows:

1. Not consuming soda for the duration of the intervention period.
2. Not consuming candy/sweets for the duration of the intervention period.
3. Not consuming white grains/rice for the duration of the intervention period, instead replacing these with complex carbohydrates such as whole grains and brown rice.
4. If consuming fruit with a meal, eating the fruit at the end of the meal to minimize glycemic spiking.

Timetable: This study, to be completed over a 3-year period, will include a net total of *n* = 34 subjects with bipolar disorder. Details of the enrollment and major test procedures are outlined in Table 3. Milestone of recruitment success is net completion of 24 persons by the end of year 2. A gross total of up to 50 subjects will be recruited to account for possible attrition.

Design: Exploratory pilot study assessing open-label ketone ester supplementation (19 g BID PO; 12.5 g active ingredient per serving) in combination with low glycemic index dietary intervention.

Inclusion criteria

To be eligible for the study, an individual must meet the following criteria:

1. Adults 18 and older, able to provide informed consent
2. Diagnosis of bipolar disorder (any subtype)

Exclusion criteria

An individual who meets any of the following criteria will be excluded from participation in the study:

1. History of traumatic brain injury
2. Evidence of large vessel stroke or mass lesion on MRI
3. History of significant gastrointestinal disease or malabsorptive disorder
4. Pregnancy or breastfeeding
5. Uncontrolled diabetes mellitus
6. History of mitochondrial disorder and/or significant uncontrolled metabolic/medical disorder
7. Active/current substance dependence (and/or consumption of two or more alcoholic beverages per day)
8. Subjects with contra-indications to MR imaging, including pacemakers or severe claustrophobia
9. Active suicidal or homicidal ideation

**Figure 1:**
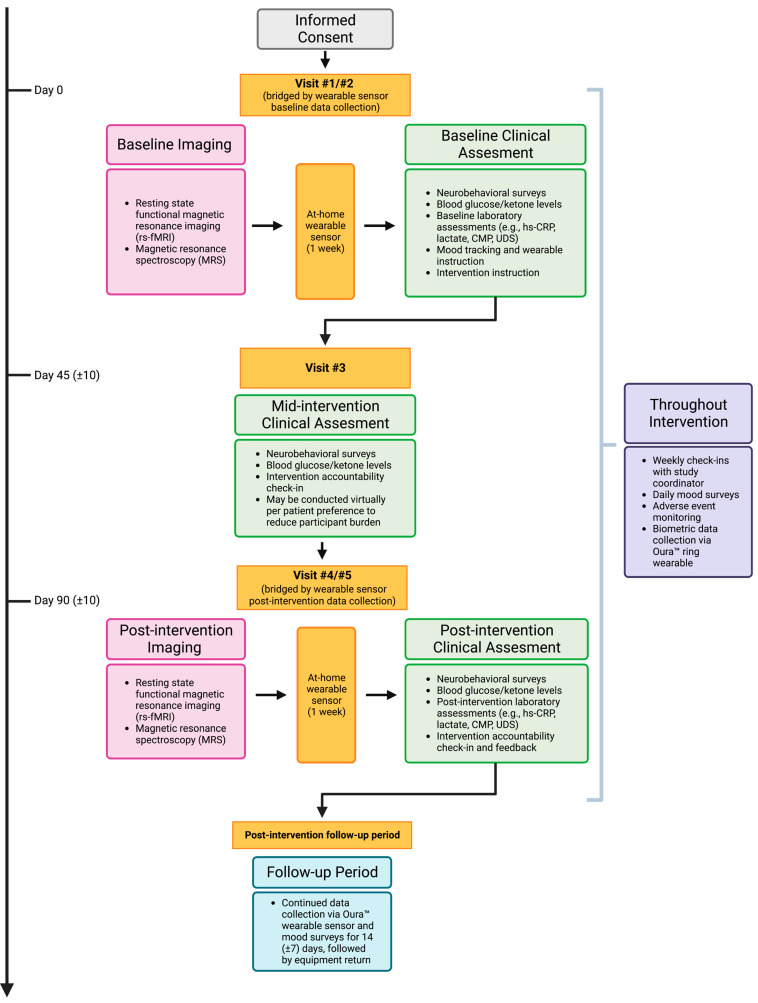
Protocol Flowchart: Visual Overview.

**Figure 2:**
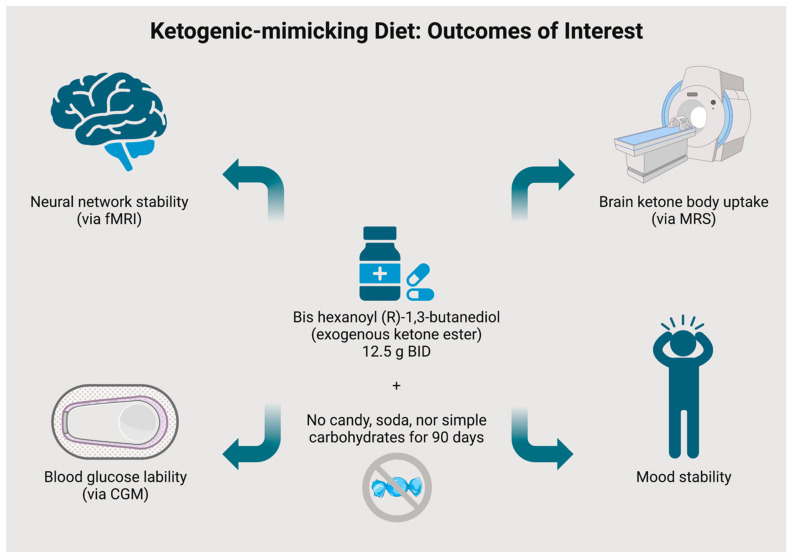
Key Outcomes: Visual Overview.

Study procedures and assessments: After obtaining informed consent and screening for study eligibility, study participants will undergo the baseline clinical assessment and imaging protocol (separate days). There will be a total of 5 visits for this pilot study. The baseline clinical assessment will include standardized assessments of global functioning and symptom severity (summarized in Table 1). Participants will also be instructed to wear a continuous glucose monitor (CGM) and a biometric device called Oura™ ring for 7 (±3) days prior to starting the intervention, allowing for assessment of baseline glycemic trends as well as sleep and activity data. They will also have their blood drawn for laboratory assessments at the pre-intervention clinical visit (assays detailed in Table 2)—blood-derived laboratory assays will be optional for participants (e.g., to accommodate participants with a strong preference to avoid blood draws or a specific phobia pertaining to blood). Upon completion of this baseline assessment, participants will initiate the 90 (±10)-day intervention (ketone ester supplementation in combination with low glycemic index dietary changes). During the intervention period, participants will be prompted to submit daily ratings of mood via the CareEvolution platform. They will also be instructed to check their blood ketone/glucose levels using a Keto-Mojo™ ketone/glucose monitor on intervention days 0 (baseline), 45 (±10) (mid-intervention), and 90 (±10) (post-intervention): measurements will be taken before supplementation (morning fasting) and two hours post-supplementation. At the mid-intervention point (day 45 ± 10 days), participants will repeat the comprehensive neurobehavioral assessment summarized in Table 1. At the end of the intervention period, they will repeat a 7–10 day CGM assessment. After completing the intervention, participants will repeat the clinical/laboratory assessments and imaging protocol (on separate days). They will continue to wear their Oura™ ring throughout the intervention period and for a 14 (±7)-day post-intervention follow-up period. Daily mood ratings will also be continued during this post-intervention follow-up period until the Oura™ wearable is returned, at which point study participation will be completed. For a visual overview of this study design, please refer to the schedule of events outlined in Table 3 and the flowchart depicted in Figure 1. A visual overview of key outcome metrics is featured in Figure 2.

**Table 3:** Study Overview.

Outcome Metric	Pre-Intervention Imaging *	Baseline Clinical Assessment *	Daily	Mid-Intervention	Post-Intervention Imaging *	Post-Intervention Clinical Assessment *
fMRI/MRS	X				X	
CGM	X	X			X	X
Oura™	X	X	X	X	X	X
YMRS		X		X		X
BDI		X		X		X
PHQ-9		X		X		X
Mood Surveys		X	X	X		X
CGI		X		X		X
GAF		X		X		X
LFQ		X		X		X
SDS		X		X		X
STAI		X		X		X
SF-36		X		X		X
Blood ketones		X		X		X
Blood glucose		X		X		X
Lab assays (see Table 2)		X				X

* For visits 1–2 and 4–5: There is no pre-set sequence for which assessment (clinical or imaging) comes first.

**Hypothesis 1**. *Neural network stability in subjects with bipolar disorder will improve after the approximately 90-day intervention involving supplementation with 19 g BID PO of ketone ester (containing 12.5 g C6 ketone di-ester per serving).*

**Hypothesis 2**. *Greater neural uptake of ketone bodies as measured via MRS will correlate with greater improvement in neural network stability.*

**Hypothesis 3**. *Greater glucose lability (glucose spikes demonstrated via CGM trends) will correlate with less favorable response to the intervention.*

**Hypothesis 4**. *Lower Glucose Ketone Index (GKI) (as measured via Keto-Mojo™ device) will correlate with more favorable response to the intervention.*

Exploratory Hypothesis 1: Neural network instability as ascertained via fMRI may correlate with mood instability as ascertained via self-reported mood surveys.

Exploratory Hypothesis 2: Ketone ester supplementation combined with low glycemic index dietary changes may correlate with improved metabolic biomarkers (e.g., hs-CRP or serum lactate).

Exploratory Hypothesis 3: Greater neural uptake of ketone bodies as measured via MRS and greater increases in blood ketone level as measured via Keto-Mojo™ device may correlate with greater improvements in mood stability and global functioning.

Impact: Positive findings in this small exploratory pilot study may support future target engagement studies of ketone ester supplementation or other ketogenic interventions in bipolar disorder.

## 4. Statistical Analysis

Mixed linear modeling will be used to assess changes in fMRI neural network stability and MRS neural uptake of ketone bodies.

As *post hoc* and only exploratory analyses, we will regress differences pre- and post-intervention in various mood/clinical, global functioning, and laboratory outcome measures.

## 5. Discussion

**Figure 3:**
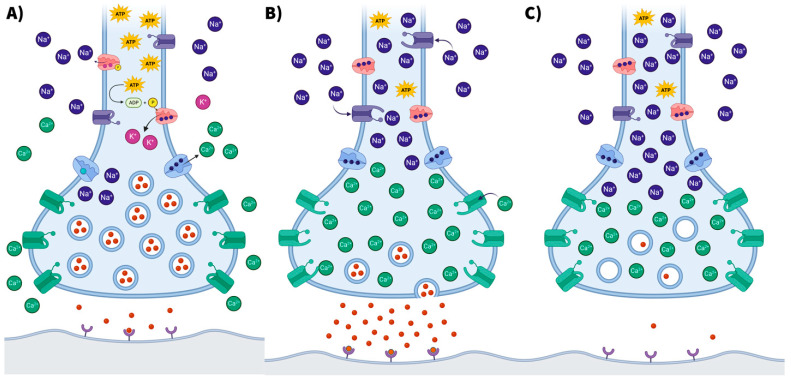
Neuronal Mechanisms Implicated in Bipolar Disorder: Hypothesized Conceptual Model. (**A**) Typical neuronal model as baseline comparison. With proper homeostatic regulation of electrochemical gradients, neuronal excitability is optimized. (**B**) Neuronal model with intracellular sodium accumulation secondary to secondary to Na^+^/K^+^ ATPase hypofunctionality. In turn, this would be expected to raise neuronal membrane potential, resulting in hyperexcitability and inappropriate target engagement (i.e., excessive neurotransmitter release from vesicles)—thus, a working mechanistic model applicable to (hypo)manic episodes. Interestingly, individuals with bipolar disorder have been observed to exhibit decreased Na^+^/K^+^ ATPase activity and intracellular sodium accumulation in a mood-related pattern [27]. Moreover, stimulants and antidepressants would be capable of converging on synaptic hyperexcitability mechanisms and are widely known to trigger (hypo)mania in susceptible individuals. (**C**) Neuronal model illustrating synaptic vesicle depletion as a potential downstream consequence of hyperexcitability and inappropriate target engagement. Synaptic vesicle depletion may result in phasic hypoactive synaptic signaling until homeostasis is restored. This provides a working mechanistic model applicable to depressive episodes that helps account for the temporal relationship commonly observed between (hypo)manic and depressive episodes.

**Figure 4:**
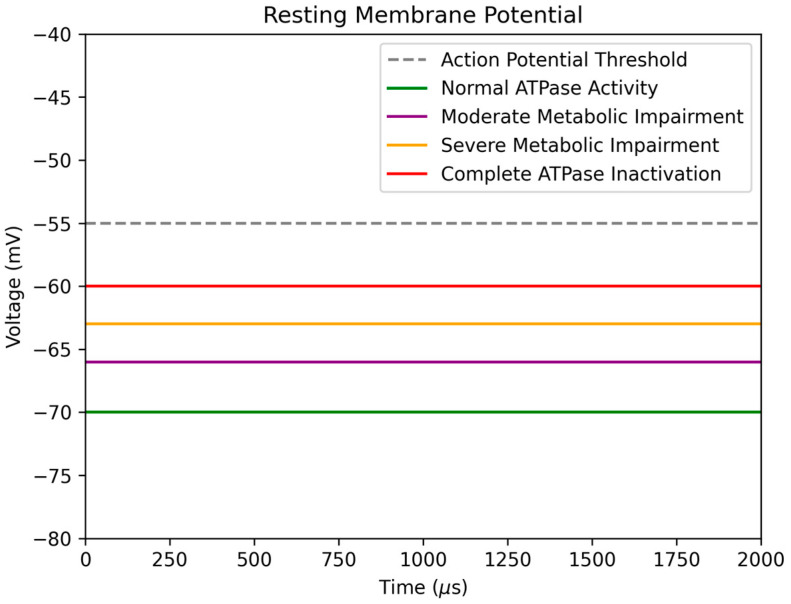
Conceptual illustration of how resting membrane potential may be altered as a function of metabolic impairment and resultant Na^+^/K^+^ ATPase hypofunctionality. With increasing severity of metabolic impairment, resting membrane potential may rise secondary to Na^+^/K^+^ ATPase hypofunctionality. In turn, this may alter neuronal excitability. As a point of reference that speaks to the delicate nature of neuronal membrane homeostasis, complete inactivation of the Na^+^/K^+^ ATPase would result in a resting membrane potential that is approximately 10 mV less electronegative compared to baseline [59].

Here, we synthesize our review of the literature into a biomechanistic rationale for the therapeutic potential of ketogenic interventions in the setting of bipolar disorder. Building on the groundwork laid by leaders in this field, we have illustrated a hypothesized conceptual model for investigation, which outlines mechanistic pathways implicated in the pathogenesis of the bipolar disorder. To help visualize the neuronal mechanisms proposed by El-Mallakh et al. [27], we have created comparative figures depicting altered neuronal excitability in the setting of Na^+^/K^+^ ATPase hypofunctionality. Figure 3A displays a typical neuronal model as a baseline comparison. Figure 3B illustrates a neuronal model with intracellular sodium accumulation secondary to Na^+^/K^+^ ATPase hypofunctionality. In turn, this would be expected to raise neuronal membrane potential, resulting in hyperexcitability and inappropriate target engagement (i.e., excessive neurotransmitter release from vesicles)—thus, a working mechanistic model applicable to (hypo)manic episodes. A potential downstream consequence of excessive neuronal excitability is synaptic vesicle depletion, which may result in phasic hypoactive synaptic signaling until homeostasis is restored. This provides a working mechanistic model for depressive episodes (illustrated in Figure 3C) that helps account for the temporal relationship commonly observed between (hypo)manic and depressive episodes. Figure 4 provides a conceptual illustration of how resting membrane potential may be altered as a function of metabolic impairment and resultant Na^+^/K^+^ ATPase hypofunctionality. Although medication side effects, sleep disruption, and lifestyle factors represent noteworthy confounders when considering potential causal relationships, higher rates of metabolic syndrome have been identified in patients newly diagnosed with bipolar disorder (odds ratio of 3.529, 95% CI 1.378–9.041, *p* = 0.009) [57]. Lower levels of ATP and phosphocreatine in the brain have also been identified in individuals with bipolar disorder [58]. These findings suggest that metabolism may play an important role in bipolar pathophysiology.

**Figure 5:**
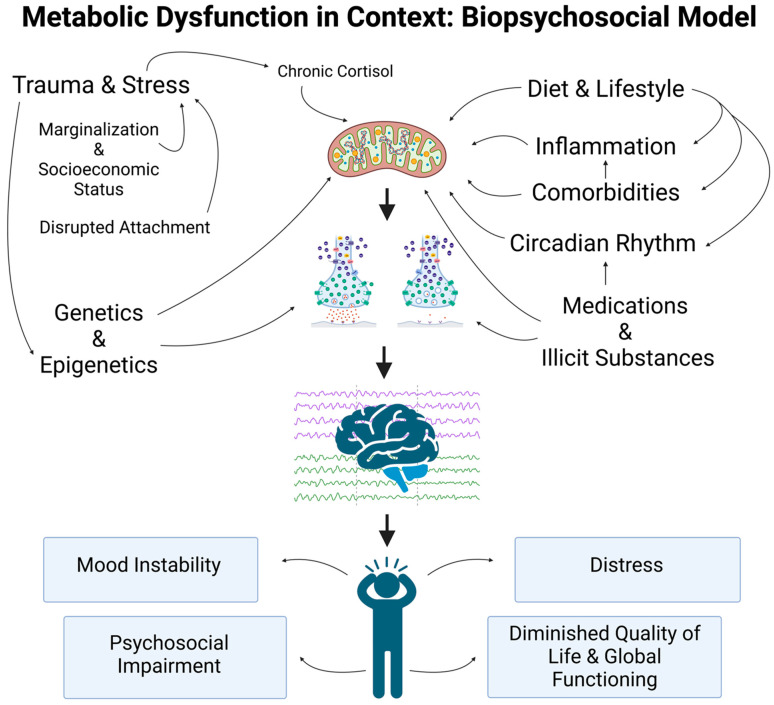
Metabolic Dysfunction in Context: Biopsychosocial Model. Importantly, metabolic dysregulation should be conceptualized within the broader context of the biopsychosocial model. There are many upstream and downstream factors integrated with metabolism, including lifestyle factors (such as nutrition, exercise, and sleep), medical comorbidities, medications, substance use, trauma, and chronic stress. Accumulating evidence supports the critical role of mitochondrial function in bipolar illness, which may help explain how altered neuronal excitability represents just one component of a broader model connecting biological and metabolic mechanisms with psychological and social risk factors [60]. As a pertinent example, stress and trauma have been linked to alterations in cortisol signaling and mitochondrial function [61]. Moreover, this figure illustrates a critical link between altered neuronal excitability and altered patterns of neural network functional connectivity, as patterns of spreading activation throughout neural networks are inherently dependent on patterns of neuronal excitability. The foundation of the image depicts how these mechanisms may ultimately culminate in suffering experienced by the patients we serve—something that should not be overlooked for the sake of mechanistic granularity.

To conceptualize altered neuronal excitability as a function of metabolic impairment—a broad term—naturally begs the question of what mechanisms may lie upstream of Na^+^/K^+^ ATPase hypofunctionality. The most intuitive consideration is energetic availability in the form of ATP given the striking energetic requirements of the Na^+^/K^+^ ATPase, though this answer is not exhaustive. Accumulating evidence supports the critical role of mitochondrial function in bipolar illness, which may help explain how altered neuronal excitability represents just one component of a broader model connecting biological and metabolic mechanisms with psychological and social risk factors [60]. As a pertinent example, stress and trauma have been linked to alterations in cortisol signaling and mitochondrial function [61]. Figure 5 provides a visual overview of how metabolic dysregulation may be conceptualized within the broader context of the biopsychosocial model. Moreover, this figure illustrates a critical link between altered neuronal excitability and altered patterns of neural network functional connectivity, as patterns of spreading activation throughout neural networks are inherently dependent on patterns of neuronal excitability. Notably, prefrontal cortex hypometabolism as measured via ^18^F-fluoro-deoxyglucose positron emission tomography (18F-FDG-PET) has been observed in the setting of bipolar disorder, suggesting that disruption of prefrontal modulatory effects may drive changes in other neural networks [62,63].

A mechanistic understanding of potential contributory pathways to bipolar illness helps inform our efforts to identify clinically useful biomarkers and novel therapeutic targets. Accumulating evidence supports a clear rationale to investigate ketogenic interventions as a potential therapeutic modality in bipolar disorder. Perhaps most pertinent from a mechanistic standpoint is the body of emerging evidence supporting the role of mitochondrial dysfunction in the pathogenesis of bipolar disorder. Ketogenic interventions are widely known to promote mitochondrial turnover and biogenesis, in addition to potentiating alterations in neuronal excitability, anti-inflammatory effects, and GABA/glutamate ratio modulation via the gut–brain axis. The authors thus recognize the potential for ketogenic interventions as an investigational therapy for patients with bipolar disorder—particularly given the outcomes achieved by the ketogenic diet in the realm of epilepsy, which support a role for modulation of neuronal excitability and improvement of mitochondrial function. With a century of research demonstrating the efficacy of therapeutic ketosis in treatment-resistant epilepsy, the pleiotropic mechanisms enacted via ketogenic interventions may also help address the complexity of treatment-resistant mental illness. While this line of reasoning is supported by limited preliminary data, further research is necessary to validate these hypotheses. This pilot study will serve to advance a mechanistic understanding of potential benefits that may be derived from ketogenic interventions in the setting of bipolar disorder.

Although this protocol focuses on four simple dietary changes to minimize glycemic spiking (summarized for reference in Table 4), the authors recognize the potential for more comprehensive low glycemic index diets (including strict ketogenic diets) and other synergistic interventions (e.g., proper sleep hygiene and exercise) to augment the effects of exogenous ketone supplementation. We propose the term ‘ketogenic-mimicking diet’ to recognize the potential for modified dietary interventions to emulate at least some of the benefits driven by more restrictive ketogenic diets. It is possible that minimizing glycemic/insulin spiking via dietary changes while promoting ketone-driven signaling mechanisms and bioenergetic availability via exogenous ketosis may bear clinical implications for the management of bipolar disorder, though more research is needed to investigate this.

In advancing our understanding of ketogenic therapies, it is important to appreciate the nuanced and pleiotropic mechanisms enacted by ketone bodies, which may vary in relative importance as a function of the clinical context. While the ‘alternative fuel’ properties of ketone bodies are considered to be particularly impactful in the setting of the brain ‘energy gap’ implicated in Alzheimer’s disease, to our knowledge there is not a single publication to date reporting a clinical response achieved solely via exogenous ketone or ketone precursor supplementation (i.e., ‘alternative fuel’ availability alone) in the setting of bipolar disorder—in contrast to clinical outcomes reported in individuals with Alzheimer’s disease [64]. This suggests the possibility that more comprehensive metabolic interventions (e.g., involving reduced glycemic intake) and/or ‘signaling’ (e.g., anti-inflammatory, epigenetic, mitochondrial, and neuromodulatory) effects of ketone bodies may bear relatively greater importance in the setting of bipolar disorder, particularly given the mechanistic overlap between bipolar disorder and seizures (as both involve complex pathophysiological underpinnings that plausibly require more than simply increasing bioenergetic availability to address) [53,64,65]. In turn, this may bear implications for the clinical utility of differing ketogenic agents and therapeutic strategies (notably, ß-hydroxybutyrate enantiomers are known to differ in predominance of ‘fuel’ vs. ‘signaling’ properties). Future research is needed to elucidate optimal ketogenic protocols and potential synergistic interventions in the setting of bipolar disorder.

It is imperative for any such protocol to respect the complexity of intersecting factors affecting metabolism. As a pertinent example serving as the impetus for our protocol, it is widely known that ketone metabolism is altered in the setting of high glycemic intake and resultant elevations in insulin signaling. Looking to the future, further research may reveal a potential role for ‘metabolic switching’ (alternating between states of energetic abundance and autophagy) as a therapeutic strategy for bipolar disorder, as this intervention has been shown to modulate GABAergic interneuron dysfunction and neuronal hyperexcitability in other clinical settings [66]. While these possibilities certainly require thorough investigation, they suggest a bright future for the emerging field of metabolic psychiatry.

## 6. Limitations

A number of limitations must be recognized for this research protocol. Importantly, this pilot study protocol does not feature a control group for feasibility and budgetary purposes. While the biasing of outcomes via potential placebo effects can be mitigated to some extent by focusing on objective biomarker outcomes, this methodology nonetheless introduces confounders that will undoubtedly need to be addressed in subsequent studies. Another key consideration for this protocol is the challenge of adherence to a dietary intervention. While adherence to the intervention will be assessed via self-report (through both regular check-ins with study coordinators and daily consumption logs), glucose level trends (ascertained via CGM data), ketone level trends (ascertained via Keto-Mojo™ data), and supplement accountability (including study coordinators measuring how much ketone ester supplement has been exhausted from containers at study completion), it must be noted that each of these adherence measures has limitations and imperfections. Finally, the authors anticipate that the effect size associated with a ‘ketogenic-mimicking diet’ will be moderate relative to the strong effect sizes reported with strict ketogenic diets. With no preexisting clinical studies investigating ketone ester supplementation combined with low glycemic index dietary changes in the setting of bipolar disorder, some educated assumptions are inherently necessary when predicting statistical power and minimum sample size. This suggests the possibility that a small pilot study may be underpowered given the uncertain magnitude of biomechanistic effects.

## Figures and Tables

**Table 1:** Neurobehavioral survey battery.

Clinical Outcome	Metric
Mania Symptomatology	Young Mania Rating Scale (YMRS)
Depression Symptomatology	Beck Depression Inventory (BDI) Patient Health Questionnaire-9 (PHQ-9)
Mood Stability	Brief daily mood surveys delivered via CareEvolution platform
Global Functioning	Clinical Global Impression (CGI) Global Assessment of Functioning (GAF) Life Functioning Questionnaire (LFQ) Sheehan Disability Scale (SDS)
State and Trait Anxiety	Spielberger State-Trait Anxiety Inventory (STAI)
Quality of Life	36-Item Short Form Health Survey (SF-36)

**Table 2:** Laboratory assessments *.

High-sensitivity C-reactive protein (hs-CRP)
Comprehensive metabolic panel (CMP)
Peripheral mitochondrial function assessment (e.g., serum lactate, WBC mitochondrial DNA copy number, mitochondrial haplotype, or Seahorse assay)
Urine drug screen (UDS)

* Lithium level monitoring may be considered for participants taking lithium due to theoretical interaction effects in the setting of ketone metabolism.

**Table 4:** Simple dietary changes to reduce glycemic spiking.

1.	Not consuming soda for the duration of the intervention period.
2.	Not consuming candy/sweets for the duration of the intervention period.
3.	Not consuming white grains/rice for the duration of the intervention period, instead replacing these with complex carbohydrates such as whole grains and brown rice.
4.	If consuming fruit with a meal, eating the fruit at the end of the meal to minimize glycemic spiking.

## Data Availability

Publicly available data from OpenNeuro repository “Protecting the Aging Brain—Diet Study” at https://openneuro.org/datasets/ds003437/versions/1.0.2 (accessed on 3 July 2023) was used for sample size calculations, which are detailed in the Supplementary Materials.

## Supplementary Materials

The following supporting information can be downloaded at: https://www.mdpi.com/article/10.3390/nu15133068/s1, Figure S1: Boxplot of participant DMN instabilities by condition; Table S1: Computed DMN instabilities by condition. References [45,67,68,69] are cited in supplementary file.
