# Supplementary material for: Ketogenic-Mimicking Diet as a Therapeutic Modality for Bipolar Disorder: Biomechanistic Rationale and Protocol for a Pilot Clinical Trial
Source: Nutrients. 2023 Jul 7;15(13):3068. doi: 10.3390/nu15133068 (PMC10346691; doi:10.3390/nu15133068)
Supplement: Supplementary file 1 [file nutrients-15-03068-s001.zip › nutrients-2467077-supplementary.pdf]

Supplementary Materials

Section 1. Sample size calculation

Sample size calculation was based on findings published by Mujica-Parodi et al. [1], which presented the first evidence for stabilizing effects of ketosis on resting state functional connectivity networks. Public dataset [2] and code (https://github.com/bbantal/PAgB_network_instability) was used for calculation of network instability across a sample of 12 participants from that study. Default mode network (DMN) was chosen for calculating instabilities due to its profound energetic demand [3], extensive association with the symptomology of bipolar disorder [4], and its intrinsic predominance during the task-free resting state conditions under which our participants will be scanned. The following set of DMN instabilities was obtained for both standard diet and 1-week ketogenic diet conditions for each of the 12 participants, with τ time constant set at 1 and time windows of 24 seconds, as in the original study [1]. See Supplementary Table S1 and Figure S1:

Supplementary Table S1. Computed default mode network (DMN) instabilities by condition

Participant	DMN Instability	Condition
**sub001**	0.6831335	Standard Diet
**sub002**	0.5992506	Standard Diet
**sub005**	0.6130102	Standard Diet
**sub019**	0.6237661	Standard Diet
**sub022**	0.4820238	Standard Diet
**sub027**	0.5731876	Standard Diet
**sub028**	0.4069916	Standard Diet
**sub031**	0.6780192	Standard Diet
**sub032**	0.4900810	Standard Diet
**sub034**	0.6041005	Standard Diet
**sub036**	0.5906136	Standard Diet
**sub038**	0.5027476	Standard Diet
**sub001**	0.3998186	Ketogenic Diet
**sub002**	0.5014335	Ketogenic Diet
**sub005**	0.4027200	Ketogenic Diet
**sub019**	0.4504665	Ketogenic Diet
**sub022**	0.3769252	Ketogenic Diet
**sub027**	0.4965531	Ketogenic Diet
**sub028**	0.5006693	Ketogenic Diet
**sub031**	0.7310587	Ketogenic Diet
**sub032**	0.4335100	Ketogenic Diet
**sub034**	0.4655478	Ketogenic Diet
**sub036**	0.5401029	Ketogenic Diet
**sub038**	0.5742642	Ketogenic Diet

Supplementary Figure S1. Boxplot of participant DMN instabilities by condition

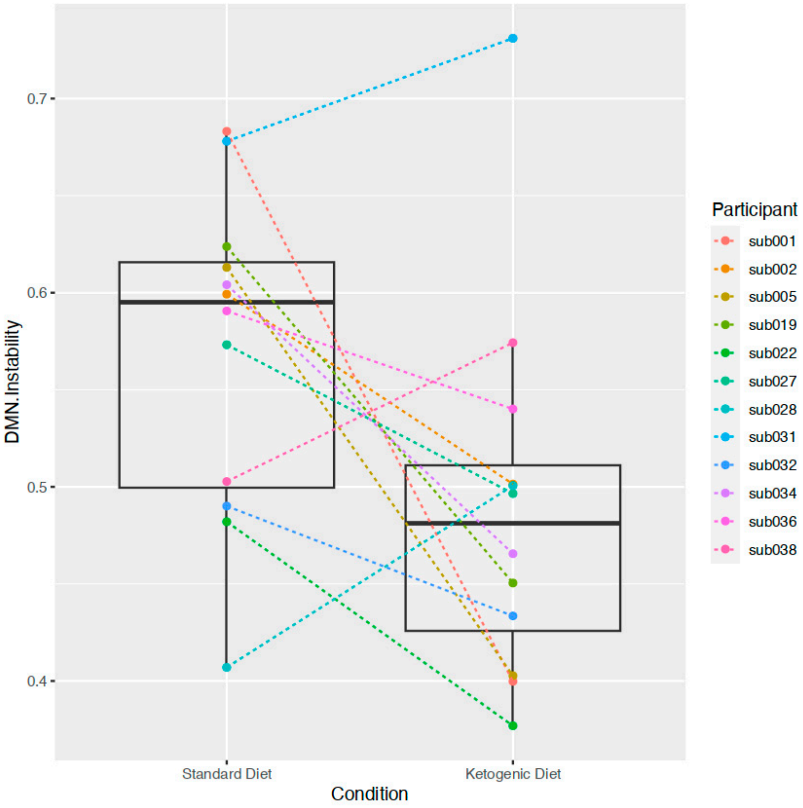

Mean difference (M_{diff}) was divided by the pooled standard deviation (S_p) of DMN instabilities between the two conditions to estimate Cohen's *d* effect size:

$$M_{diff} = M_{ket} - M_{std}$$

$$S_p = \sqrt{\frac{(S_{ket}^2 + S_{std}^2)}{2}}$$

$$d = \frac{M_{diff}}{S_p}$$

The resulting Cohen's *d* estimate was calculated to be -0.90, generally considered a strong effect. Lastly, *R* programming language package *pwr* (https://github.com/heliosdrm/pwr) was used to estimate paired samples *t*-test sample size at a significance level of 0.05, power of 0.8, and computed effect size estimate of -0.90. The resulting estimated minimal sample size was computed at 12 participants. However, this sample size is based on strict ketogenic diet intervention data, which might yield a greater effect on DMN instability than our proposed ketogenic-mimicking diet. Assuming that the effect of a ketogenic-mimicking diet is moderate as compared to the strong effect of a strict ketogenic diet, we reduced our expected effect size to -0.5, which yielded an estimated sample size of 34 participants.

References

[1] L. R. Mujica-Parodi *et al.*, “Diet modulates brain network stability, a biomarker for brain aging, in young adults,” *Proc. Natl. Acad. Sci.*, vol. 117, no. 11, pp. 6170–6177, Mar. 2020, doi: 10.1073/pnas.1913042117.

[2] L. R. Mujica-Parodi *et al.*, “Protecting the Aging Brain - Diet-Study.” Openneuro, 2021. doi: 10.18112/OPENNEURO.DS003437.V1.0.2.

[3] M. E. Raichle and A. Z. Snyder, “A default mode of brain function: A brief history of an evolving idea,” *NeuroImage*, vol. 37, no. 4, pp. 1083–1090, Oct. 2007, doi: 10.1016/j.neuroimage.2007.02.041.

[4] B. Bi, D. Che, and Y. Bai, “Neural network of bipolar disorder: Toward integration of neuroimaging and neurocircuit-based treatment strategies,” *Transl. Psychiatry*, vol. 12, no. 1, p. 143, Apr. 2022, doi: 10.1038/s41398-022-01917-x.
